# Increasing incidence of childhood leukaemia: a controversy re-examined

**DOI:** 10.1038/sj.bjc.6603946

**Published:** 2007-08-21

**Authors:** A Shah, M P Coleman

**Affiliations:** 1 Non-communicable Disease Epidemiology Unit, Department of Epidemiology and Population Health, London School of Hygiene and Tropical Medicine, Keppel Street, London WC1E 7HT, UK

**Keywords:** childhood leukaemia, incidence, mortality, trends

## Abstract

We provide evidence of a gradual increase in the incidence of childhood leukaemia over the twentieth century from examination of trends in both incidence and mortality in England and Wales. We conclude that much of the recorded increase is likely to be real.

In Europe, childhood lymphoid leukaemia incidence (including acute lymphoblastic leukaemia (ALL)) increased significantly by an average of 1.4% per year during 1970–1999 (Steliarova-Foucher *et al*, 2004). In England and Wales, the increase in ALL has been attributed to the ‘common’ precursor B-cell subtype (Feltbower *et al*, 2001; Kroll *et al*, 2006). Debate has arisen over whether the increase is artefactual (Leukaemia Research Fund, 2004; Adamson *et al*, 2005) or real (Steliarova-Foucher *et al*, 2004, 2005).

Fewer than 5% of children diagnosed with leukaemia in England before the early 1960s survived more than 5 years after diagnosis (Thames Cancer Registry, 1994), and mortality trends before 1960 may thus be considered a reasonable proxy for incidence. We have therefore examined all the available data on incidence and mortality trends for childhood leukaemia in England and Wales over most of the twentieth century.

Leukaemia is the commonest cancer in children (0–14 years), representing a third of all malignancies (Parkin *et al*, 1998). Incidence rates increase to a peak around age 3–4 years and then decline (Swerdlow *et al*, 2001). Some 400 children are diagnosed in England and Wales each year, and about 100 die of it. Four out of five cases of leukaemia in children are ALL, and the remaining almost all acute myeloid leukaemia (AML).

## MATERIALS AND METHODS

Data on childhood leukaemia incidence and mortality in England and Wales up to the year 2000 were used to examine trends by age (under 1 year, and 1–4, 5–9, 10–14 years), sex and 5-year period. Mortality data for 1911–2000 and incidence data for 1971–1990 were obtained from the Office for National Statistics (ONS) (Office for National Statistics, 1998, 2003). Additional cancer registration data for the period 1991–2000 were obtained separately from ONS. Annual incidence and mortality rates for children aged 0–14 years were adjusted to the world standard population (Parkin *et al*, 1992). Variance-weighted least-squares linear regression was used to estimate the average quinquennial change in incidence and mortality rates.

Mortality data held by ONS span the whole of the twentieth century. Before 1959, however, the only data available are those published in the Registrar General's Annual Reviews. Computerised information on individual deaths has only been available since 1959. These data have been published on CD-ROM (Office for National Statistics, 2003).

Death registrations derived from medical certificates of cause of death are coded at ONS to the World Health Organisation's (WHO) International Classification of Diseases (ICD). The ICD underwent 10 revisions during the twentieth century, reflecting successive advances in medical knowledge and disease classification. The first version, in force during 1901–1910, did not include a separate category for leukaemia, so it is impossible to estimate national mortality rates for childhood leukaemia before 1911. The ICD codes for leukaemia data used in the analyses are given in Table 1.

The ONS has coordinated cancer registration nationally in England and Wales since 1971 through a network of population-based regional cancer registries in England and a national cancer registry in Wales (Office for National Statistics, 2004a).

## RESULTS

**Figure 1:**
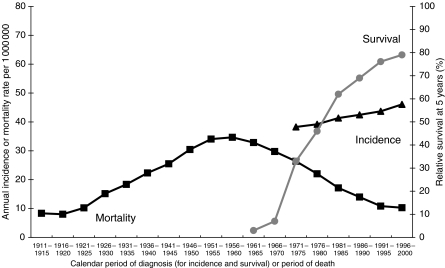
Trends in leukaemia incidence, survival and mortality in children (0–14 years), England and Wales, 1911–2000. Incidence and mortality rates per million person-years for England and Wales (Office for National Statistics, 1998, 2003). Five-year relative survival rates (%) for children diagnosed in South-East England 1960–1970 (Thames Cancer Registry, 1994), in England and Wales during 1971–1990 (Coleman *et al*, 1999) and in Great Britain 1991–2000 (Shah, 2005).

Childhood leukaemia mortality rates increased four-fold from 8.4 per million person-years in 1911–1915 to reach 34.7 by the late 1950s (Figure 1). The decline in mortality over the second half of the twentieth century to 10.3 per million person-years has been similarly impressive.

Leukaemia mortality peaked earlier for children under 5 years of age (1941–1955) than for older children (1956–1970). The 5-yearly decline in childhood leukaemia mortality was significant for each age group over the period 1971–2000. Peak mortality varied widely by age, from 23.4 per million for children aged 10–14 years to 51.8 at ages 1–4 years (Table 2). The increase and almost symmetric decrease in mortality is particularly marked in children aged 1–4 years, with a peak of 51.8 deaths per million person-years during 1951–1955, declining by 80% to 10.6 by 1996–2000.

Incidence rose by 20% between the early 1970s and the end of the century (Figure 1), an increase of 4% every 5 years. Both incidence and mortality were at least 15% higher in boys than girls throughout the twentieth century. Incidence at ages 1–4 years was more than double that of any other age group (Table 3). The average 5-yearly increase in incidence over the period 1971–2000 was significant in all age groups, but the overall increase was much larger among children under 5 years (46% in infants, 24% in children aged 1–4 years) than in older children (12–15%).

## DISCUSSION

The divergence between rising incidence and falling mortality is remarkable, but it reflects the great improvement in 5-year survival, from less than 5% in the early 1960s (Thames Cancer Registry, 1994) to almost 80% by the end of the 1990s (Coleman *et al*, 1999; Shah, 2005). Since survival was so low before the early 1960s, it seems likely that mortality from 1911 up to the 1950s provides a reasonable proxy for incidence trends. Taken together with the directly observed incidence trends from the 1970s to the late 1990s, these data strongly suggest a century-long increase in childhood leukaemia, particularly in children under 5 years of age.

Similar trends for childhood leukaemia have been observed in the United States, with an increase in mortality until the 1960s. The subsequent decline in mortality, resulting from improvements in treatment, has been seen world-wide (Fraumeni and Miller, 1967; Coleman *et al*, 1993; Smith *et al*, 1999). Incidence has increased significantly in Europe and in the United States since the 1970s, which is essentially attributable to ALL (Coleman *et al*, 1993; Smith *et al*, 1999; Xie *et al*, 2003; Steliarova-Foucher *et al*, 2004). However, the incidence of ALL and AML has been stable in the Nordic countries between 1982 and 2001 (Hjalgrim *et al*, 2003).

The childhood peak at 2–4 years that began to develop in the 1920s in England and Wales developed later in other nations, for example in the 1940s in US Caucasians and in the 1960s in Japan and in US non-Caucasians, but it has not been seen in some developing countries (Fraumeni and Miller, 1967; Greaves *et al*, 1985). Differences in leukaemia incidence, most apparent at ages 2–3 years, have been observed between Caucasian and African-American children in the United States, and between children in western and eastern Europe (Doll, 1989; Smith *et al*, 1999; Steliarova-Foucher *et al*, 2004). The development of this age peak is the main component of the overall increase in leukaemia incidence in England and Wales and other countries over the twentieth century.

The cause of childhood leukaemia remains unknown. It has been suggested that a leukaemic exposure arose in developed countries in the early part of the twentieth century possibly associated with improved living standards (Doll, 1989). Although improvements in case-finding and in the accuracy of death certification in England and Wales are considered to have had an impact on childhood leukaemia mortality, particularly during the early part of the century, the continued increase in mortality into, and beyond, the 1950s indicates a real increase in incidence (Court Brown and Doll, 1961; Doll, 1989; Swerdlow *et al*, 2001). More complete ascertainment by cancer registries has also been proposed (Adamson *et al*, 2005), and while this may have had some impact in the early 1970s, under-ascertainment of childhood cancers has been estimated at less than 5% during 1971–1984 (Hawkins and Swerdlow, 1992). It also seems implausible that any systematic underreporting of leukaemia would have been restricted to children under 5 years, and particularly to those with the precursor B-cell subtype of ALL.

It has been hypothesised that, in the first few decades of the twentieth century, a fatal infection (e.g. pneumonia) attributable to pre-leukaemic immunosuppression would have killed a proportion of children before the leukaemia became obvious. After the introduction of antibiotics, the effect of fatal infections in ‘masking’ leukaemia would have declined, leading to the observed increase in leukaemia incidence (Stewart and Kneale, 1969). A state-wide study in Colorado during 1941–1959, however, concluded that the severity and often fatal outcome of childhood leukaemia would usually have resulted in hospitalisation, and that few cases would have remained undiagnosed there (Githens *et al*, 1965).

Even if fatal infections may have had some impact on ‘masking’ leukaemia during the first half of the century, their involvement cannot explain the increase in incidence of childhood leukaemia during 1971–2000. Infant mortality (in the first year of life) fell from 18 in 1971 to 6 per thousand live births in 2000 (ONS, 2004b). If all other causes of death up to age 5 years are ignored, this would allow some 12 000 children per million live births (18 minus 6 per 1000) each year to reach age 5 years who would not previously have done so. Leukaemia incidence in children aged 1–4 years increased from 64.5 per million person-years during 1971–1975 to 79.8 during 1996–2000, an increase of 15.3 cases per million. The incidence of leukaemia in the 12 000 ‘extra’ children per million per year who would previously have died in infancy would have to have been 1275 per million person-years, some 20 times higher than in the rest of the population, in order for the overall decline in infant mortality in the period 1971–2000 to account for the overall increase in leukaemia incidence.

In 1940, the WHO rule for selecting the underlying cause when more than one cause is mentioned on the death certificate changed, with the introduction of ICD-5: this resulted in a 3% reduction in leukaemia mortality at all ages (Swerdlow *et al*, 2001). Similarly, aleukaemia, previously defined as lymphadenoma or agranulocytosis, was included with leukaemia from 1950 (ICD-6). These shifts would have caused small changes in the number of childhood deaths coded to leukaemia, but cannot explain the increase over a period of 40 years.

The above explanations seem unlikely to explain fully the long-term changes in mortality and incidence, particularly since the disparities in trends between girls and boys have been broadly consistent over the century. We are forced to conclude that the increase in incidence is largely real.

## Figures and Tables

**Table 1:** International classification of disease codes for leukaemia

**ICD revision**	**Mortality**	**Period in use for mortality**	**Incidence**	**Period in use for incidence**
2	53A	1911–1920	—	
3	65A	1921–1930	—	
4	72A	1931–1939	—	
	72B	1935–1939	—	
5	74A and 74B	1940–1949	—	
6	204	1950–1957	—	
7	204	1958–1967	—	
8	204–207	1968–1978	204–207	1971–1978
9	204–208	1979–2000	204–208	1979–1994
10	—	—	C91–C95	1995–2000

**Table 2:** Childhood leukaemia mortality rate per million person-years by sex and age, England and Wales, 1911–2000

	**Period of death**																		**Average quinquennial change (1971–2000)**		
	**1911–1915**	**1916–1920**	**1921–1925**	**1926–1930**	**1931–1935**	**1936–1940**	**1941–1945**	**1946–1950**	**1951–1955**	**1956–1960**	**1961–1965**	**1966–1970**	**1971–1975**	**1976–1980**	**1981–1985**	**1986–1990**	**1991–1995**	**1996–2000**	**Time trend^a^**	**95% confidence interval**	
All	8.4	8.0	10.2	15.2	18.4	22.3	25.5	30.4	34.1	34.7	32.9	29.7	26.4	22.0	17.1	14.0	10.9	10.3	−3.8	−3.8	−3.8
																					
*Sex*																					
Male	10.3	9.8	12.5	18.0	20.4	26.0	30.4	33.6	37.0	38.3	36.1	32.4	29.3	26.8	19.6	15.4	13.5	11.6	−3.9	−3.9	−3.9
Female	6.4	6.2	8.0	12.3	16.3	19.2	20.5	27.2	31.9	30.9	29.4	27.0	23.3	17.0	14.6	12.6	8.1	8.9	−3.2	−3.2	−3.2
																					
*Age (years)*																					
<1	9.9	10.8	11.6	17.4	19.7	28.4	29.4	25.1	29.0	23.2	24.8	19.4	18.8	13.8	12.7	8.9	9.5	6.4	−2.2	−3.0	−1.3
1–4	11.5	10.2	13.4	21.9	25.5	33.6	36.0	46.8	51.8	48.5	43.8	37.9	30.6	21.7	16.4	14.8	9.4	10.6	−3.7	−4.2	−3.1
5–9	7.5	7.0	9.5	12.6	16.2	18.4	22.1	26.3	29.7	35.6	34.7	30.2	30.9	27.2	18.7	13.6	12.2	10.0	−4.1	−4.6	−3.7
10–14	5.5	6.1	7.3	10.3	12.8	13.0	17.2	19.1	21.3	22.0	21.3	23.4	18.9	18.9	17.4	15.0	11.2	11.3	−1.8	−2.3	−1.4

a Average quinquennial change in the mortality rate per million person-years between 1971–1975 and 1996–2000.

Mortality rates are age-standardised to the world standard population.

**Table 3:** Childhood leukaemia incidence rate per million person-years by sex and age, England and Wales, 1971–2000

	**Period of diagnosis**						**Average quinquennial change (1971–2000)**		
	**1971–1975**	**1976–1980**	**1981–1985**	**1986–1990**	**1991–1995**	**1996–2000**	**Time trend^a^**	**95% confidence intervals**	
All	38.3	39.2	41.3	42.5	43.7	46.1	1.5	1.5	1.5
									
*Sex*									
Male	43.7	43.4	44.9	45.8	49.4	49.8	1.3	1.3	1.3
Female	32.6	34.7	37.6	39.0	37.8	42.2	1.7	1.7	1.7
									
*Age (years)*									
<1	25.5	25.3	29.2	33.2	32.9	37.2	2.4	1.0	3.8
1**–**4	64.5	66.6	69.0	72.6	72.9	79.8	2.8	1.7	3.8
5**–**9	32.0	31.6	33.7	32.6	34.4	35.8	0.8	0.1	1.4
10**–**14	20.7	22.1	23.5	23.7	25.7	23.9	0.8	0.3	1.3

a Average quinquennial change in the incidence rate per million person-years between 1971–1975 and 1996–2000.

Incidence rates are age-standardised to the world standard population.
